# Turning a blind eye: The struggle to inhibit attention towards unexpected negative emotions

**DOI:** 10.3758/s13415-026-01415-3

**Published:** 2026-03-18

**Authors:** Philip T. Chalk, Josef J. Ormsby, Derek H. Arnold, Alan J. Pegna

**Affiliations:** School of Psychology, The University of Queensland, Level 3, McElwain Building (24A), St Lucia, Queensland, 4067 Australia

**Keywords:** Attentional capture, Fearful faces, Expectation, Visual search, P_D_

## Abstract

We investigated whether expectations modulate the attentional capture of fearful and happy face distractors in a visual search task. Twenty-eight participants were instructed to locate a face surrounded by a color singleton and subsequently discriminate its gender. On some trials, the target was displayed concurrently with an irrelevant happy or fearful distractor. At the beginning of each block, instructions informed participants of the probability with which a happy or fearful distractor could occur. We measured lateralized event-related potentials time-locked to the irrelevant distractors and found that attentional suppression, indexed by the distractor positivity (P_D_), was only present for fearful faces when they were unexpected. No voltage differences were found for expected/unexpected happy or expected fearful face distractors. These findings suggest that facial expressions affiliated with threat have a high attentional priority due to their emotional value but only when they are unexpected; if the presence of a threat is expected, the need for attentional capture—and thus attentional suppression—is mitigated. This raises questions regarding the assumed priority of threat in visual processing and the important role of an individual’s expectations when interacting with threat in the environment.

**Supplementary Information:**

The online version contains supplementary material available at 10.3758/s13415-026-01415-3.

## Introduction

Threat-related facial expressions are thought to be acutely salient as they likely aid an organism’s survival; the rapid (if crude) processing of threat could provide an organism with precious milliseconds to react and take appropriate circumventing action (Carretie, 2014; Öhman et al., 2010). Indeed, the *threat capture hypothesis* posits that threatening faces are processed rapidly compared to nonthreatening faces and automatically grab attention (Öhman & Mineka, 2001). Typically, this in reference to angry faces, which are direct signals of threat to the individual (i.e., angry superiority effect; Hansen & Hansen, 1988). Fearful faces, on the other hand, are threat-related and may indirectly signal threat in our environment that attracts our attention (Pourtois et al., 2004; Qiu et al., 2023). Indeed, research has demonstrated that the appearance of a fearful face leads to an increase in arousal and more generalised increase in vigilance and attention (Becker, 2009; Berggren & Derakshan, 2013). Visual search designs have been put forward to test the automatic attentional capture of threat, as attentional capture should lead to quicker and more accurate detection when threat is task-relevant, and lead to a disrupted search—exhibited through an increase in reaction time (RT)—when threat is task-irrelevant distractors. Thus far evidence has suggested otherwise: happy faces can often be detected faster and with greater accuracy compared with threatening faces when emotions are task-relevant (Becker et al., 2011; Calvo & Nummenmaa, 2008), and no behavioural differences are found between happy and threat-related faces when emotions are task-irrelevant distractors (Burra et al., 2016; 2017; Hodsoll et al., 2011; Huang et al., 2011). Though a bias towards happy faces could be driven by methodological issues, as single-target visual search tasks are extremely sensitive to basic visual features (Treisman & Gelade, 1980), with evidence suggesting that salient features, such as the ubiquitous smile of a happy face, facilitates search outside the affective characteristic of those features (Calvo & Nummenmaa, 2008).

Despite the lack of behavioural evidence, electrophysiological data may provide evidence in favour of the threat-capture hypothesis. The N2-posterior-contralateral (N2pc) and distractor positivity (P_D_) are event-related potential (ERP) measures that respectively index attentional capture and suppression (Luck & Hillyard, 1994a; Eimer, 1996; Sawaki & Luck, 2010). The N2pc is characterised as a negative deflection recorded by posterior electrodes contralateral (vs. ipsilateral) to the target, usually appearing 180–280 ms poststimulus, with a maximal amplitude at PO7/PO8 (Corriveau et al., 2012; Dell'Acqua et al., 2006; Luck & Hillyard, 1994a, b). Whilst P_D_ has the same scalp distribution, less consensus has been achieved regarding its time course. It is thought to occur at either the same time-window as the N2pc (Barras & Kerzel, 2017; Hickey et al., 2009) or after the N2pc (Kiss et al., 2012). Additionally, distracting stimuli may elicit a P_D_ without an N2pc (Burra et al., 2017; Zhang et al., 2024), especially when stimuli are presented for short durations (e.g., 200 ms; Kiss et al., 2012), which is indicative of direct suppression.

N2pc amplitudes are often reported to be more negative for threat-related faces compared with happy or neutral faces when emotions are bilateral targets (see Feldmann-Wustefeld et al., 2011; Weymar et al., 2011, but also Burra & Kerzel, 2019), suggesting that greater attentional capture towards threat-related faces. Furthermore, when emotions are processed as task-irrelevant bilateral distractors, Ikeda et al. (2013) found an N2pc for fearful faces under conditions of low and high task-load, highlighting that attentional capture is not dependent on the individual’s top-down attentional capacity. Additionally, in visual search arrays where facial expressions are distractors that appear laterally and the target at the vertical midline, the N2pc appears in response to angry but not happy faces when the faces are schematic (Burra et al., 2016), and a P_D_ appears in response to angry faces but not happy faces when the faces are naturalistic (Burra et al., 2017). Thus, electrophysiological data suggest that attentional capture (Burra et al., 2016; Ikeda et al., 2013; Feldmann-Wustefeld et al., 2011; Weymar et al., 2011) and distractor suppression (Burra et al., 2017) are greater for threat-related facial expressions compared with nonthreatening counterparts, likely as the former are more salient (Burra et al., 2016, 2017).

Although arguably the value of a threat changes depending on whether its appearance can be expected. In support of this view, Sege et al. (2015) found that prior knowledge regarding upcoming picture content specifically attenuates defensive reactions to aversive events. Yet in visual search tasks, research suggests that expectations do not facilitate the detection of threating targets (e.g., spiders), but do facilitate detection of nonthreatening targets (e.g., birds; Aue et al., 2016, 2019; Abado et al., 2023). As such, it has been proposed that expectations produce no attentional bias towards threat as greater resistance to expectancy safeguards the individual from *not* detecting a threat in an emergency (Aue et al., 2019; Abado et al., 2023). However, our previous research has demonstrated that pre-attentive encoding of spatially unattended (and irrelevant) threat-related faces is qualified by their probability, and this did not extend to happy facial expressions (Chalk & Pegna, 2024). Specifically, the N170 was enhanced for spatially unattended threat-related faces compared to happy faces, but only when they appeared at a low probability. This opposes research by Aue et al. (2019) and suggests that expectations affect the early encoding of threat-related facial expressions *uniquely*.

However, it is important to consider that the task-relevancy of the threat varies between these studies. In research by Aue et al. (2019), participants must actively distinguish threatening from non-threatening stimuli (e.g., spiders from birds) to succeed in the task. In such cases, predictions may be unnecessary to avoid situations where an unexpected threat goes unnoticed (Aue et al., 2016, 2019; Abado et al., 2023). However, when a threat is irrelevant to the individual’s task, predictions may help with the suppression of threat through the mechanism of feature-based expectations (Summerfield et al., 2006). Specifically, expectations of certain stimulus features—i.e., features that signify threat—may engage an adaptive gain-control mechanism that allows limited resources to efficiently suppress those features, especially when those features are salient, as are with fearful faces (Öhman & Mineka, 2001). Yet, when those features are unexpected, they are informative and thus engender prioritized processing and attention capture (Duncan & Humphreys, 1989; Itti & Baldi, 2009). Congruent with this interpretation, feature and location regularities for distractor singletons assist with distractor inhibition, as larger RTs are found for low-probability conditions compared with high-probability conditions (Stillwell et al., 2019; Wang & Theeuwes, 2020), or random versus constant regularities (Gaspelin & Luck, 2018; van Moorselaar et al., 2021), suggesting greater distraction possibly from attentional capture—in unexpected conditions. However, this attentional capture likely depends on the saliency of stimulus features, as we demonstrated that enhanced early ERPs were specific to unexpected threat-related and not happy facial expressions (Chalk & Pegna, 2024). Though to the best of our knowledge, no research has explored the effect of feature-based expectations on the attentional capture of emotional distractors.

As such, we investigated whether the attentional capture/suppression of fearful facial expressions is qualified by expectations in a visual search task and compared this to happy facial expressions. Participants were instructed to first locate a face surrounded by a color singleton and subsequently to discriminate the gender of the face; consequently, facial expressions were task-irrelevant. On some trials, one of the remaining faces conveyed an emotional expression that was expected or unexpected. We measured lateralized ERPs to the distractor when it appeared laterally and the target appeared on the vertical midline. Because the vertical target is equally represented in both hemispheres, differences in the lateralized ERPs reflect only the processing of the distractor. Importantly this spatial configuration enabled us to compare the attentional processing of nonthreatening (happy) and threat-related (fearful) distractor expressions when they were expected (high frequency) and unexpected (low frequency). Behaviorally, we expected to find longer RTs in the distractor-present compared with distractor-absent condition, and a difference between expected and unexpected facial expressions, but no difference between the type of facial expression. With respect to EEG data, we expected to find a larger N2pc/P_D_ amplitude for threat-related faces compared to happy faces, and this effect would be enhanced when threat-related faces were unexpected, aligning with our previous research (Chalk & Pegna, 2024).

## Materials and methods

### Participants

Thirty students from the University of Queensland were recruited for the study and were compensated with course credit. Participants had normal or corrected-to-normal vision and were all right-handed (as assessed by the Edinburgh Handedness Inventory; Oldfield, 1971). Two participants were removed: Participant 17 was removed due to experimental error and Participant 24 was removed they had less than 50 trials for the unpredicted condition after ocular artefact rejection - typically 40–80 trials are necessary for adequate signal-to-noise ratio (Thigpen et al., 2017). The final sample included 28 participants with an age range from 17–50 years (*M*_age_ ± *SD*_*age*_ = 20.6 ± 6.6; 4 males). A similar design by Burra et al. (2017) found effects with 24 participants, whilst we found an interaction between expectation and facial expressions with 22 participants (Chalk & Pegna, 2024). Additionally, based on the effect size reported by Burra et al. (2016; Cohen’s d = 0.83), our sensitivity analysis suggests that the current study design yielded a statistical power of 0.98, indicating a high likelihood of detecting similar effects. The study was approved by the Human Research Ethics Committee of The University of Queensland (Application ID: 2024/HE000547). All participants provided written informed consent prior to experimentation.

### Stimuli

**Fig. 1:**
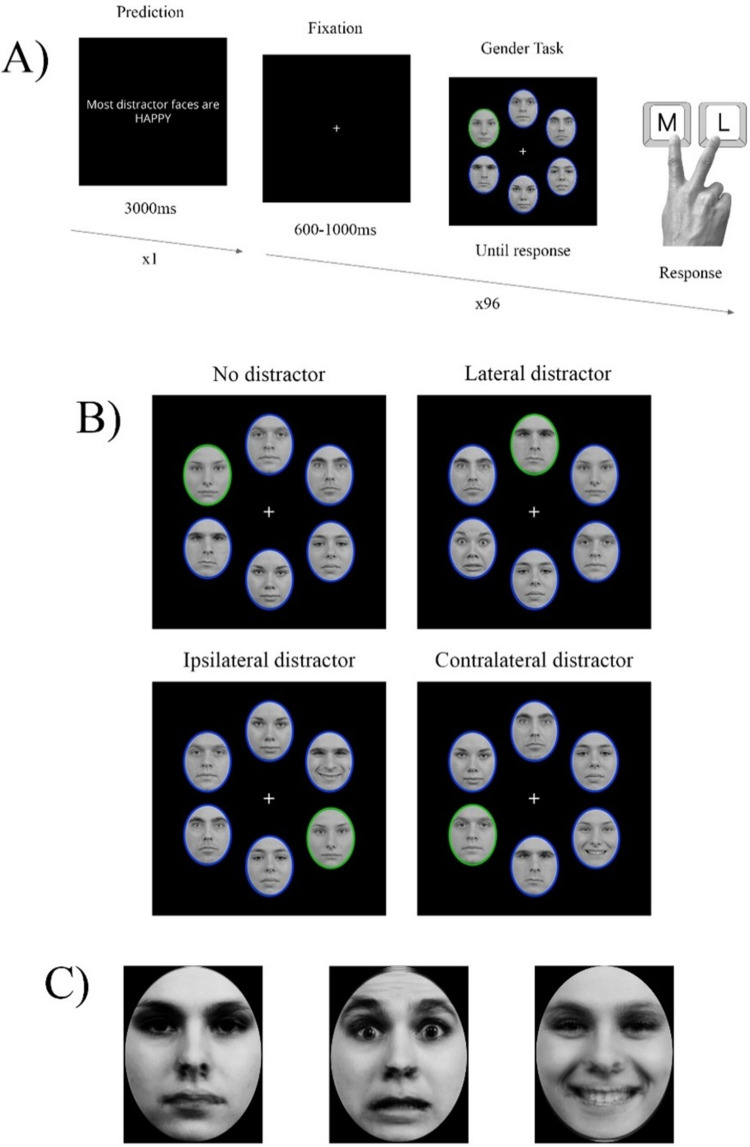
**A** Each block contained one written cue and 96 trials. Each trial consisted of a fixation cross and the gender task. **B** Illustrations of the four different face configurations. 1/3 of trials featured a lateral target with no distractor, 1/3 featured a midline target with a lateral distractor, 1/6 of trials featured a lateral target with an ipsilateral distractor and 1/6 of trials featured a lateral target with a contralateral distractor. No EEG analysis was performed on the ipsilateral and contralateral distractor conditions; rather, these conditions ensured that participants would not develop associations between the location of the target and distractor presence (**C**) Averaged neutral, fearful and happy facial expressions from left to right

Stimuli were presented on a 24-inch ASUS LCD monitor (model VG248QE; refresh rate: 144 Hz; resolution: 1920 x 1080 pixels) placed 70 cm away from the participants’ eyes. Participants used a Dell KB522p keyboard and Dell M-UAV-DEL8 mouse to enter their responses. PsychoPy (v2024.1.4) was used to present stimuli and record behavioural data on a Dell Optiplex 9020 computer running Microsoft Windows 7 Enterprise, 64-bit. Face stimuli were obtained from the Karolinska Directed Emotional Face Database (Goeleven et al., 2008). Face stimuli came from six individuals (three females, three males) with three expressions each (neutral, happy, and fearful) for a total of 18 faces. A different set of 18 faces were used in practice trials. As shown in Fig. 1, six faces were presented at 6° of eccentricity on a black background. The face stimuli were cropped within an oval of 3° horizontal and 3.8° vertical eccentricity to maintain relevant features (i.e., forehead, eyebrows, eyes, mouth, and chin). Images were rendered black-and-white with Photoshop 2021 and were equated for across luminance using the SHINE Toolbox on Matlab (Willenbockel et al., 2010). Any text presented at the center of the screen was Times New Roman, 12-point font size.

### Apparatus and procedure

Participants were seated in a dark room, and a chin rest was fixed to a comfortable height. Each trial began with a grey fixation cross on a black background, displayed for a random interval between 600 and 1,000 ms. Next, participants performed a speeded gender identification task. An array of six faces (three males, three females), were randomly presented and evenly spaced in a circle around fixation (Fig. 1). The target face could be identified by the unique colour of its border (either blue amongst green or vice versa), and participants reported the gender of the target face. All responses were made with the right hand, pressing one of two keys (M = man, L = lady). Incorrect responses were met with the word “Incorrect,” displayed for 200 ms. Keyboard responses signalled the end of one trial and the beginning of another. Each block consisted of 96 trials.

At the beginning of each block, a cue appeared for 3,000 ms, which indicated the likeliness of facial expressions in the distractor face. Cues either stated, “Most distractor faces are HAPPY” or “Most distractor faces are FEARFUL.” For the former cue, 75% of distractor faces in that block would be happy, and 25% would be fearful, and vice versa for the latter cue. Participants were instructed that emotional distractors would be present in some trials and to ignore them and focus on the gender task. Only one distractor face was ever presented at a time, and it was never the target.

Prior to experimental trials, participants completed five practice trials to verify they understood the task and that their eyes did not move from fixation. There were 20 experimental blocks in total, lasting 3 min each. Participants rested between blocks for as long as they needed. After block 10, participants had a forced 1-min minimum break. Lastly, participants rated each of the 18 experimental faces on emotional valence and intensity using a continuous scale (respectively from −100 to +100, from highly negative to highly positive, and 0–200, from no emotion to strong emotion, rescaled to 0–100).

### Electrophysiological recording and analysis

Continuous active EEG was recorded at 1024 Hz via a 24-bit, 64 channel BioSemi ActiveTwo system with a resolution of 31.25 nV (± 262 mV recording range). The 64 Ag/AgCl scalp electrodes were placed according to the internal 10–20 system location using a Waveguard nylong EEG cap for electrode placement. Recordings were referenced to the CMS/DRL electrodes. Horizontal/vertical eye movements and blinks were recorded using two electrooculographic (EOG) electrodes placed at the outer canthi and vertically below the right eye.

Pre-processing of the EEG data was performed with BrainVision Analyzer 2.0 (BrainVision Analyzer 2.0, Brain products GmbH). Individual electrodes that produced either flatline signals or sustained noise throughout the experiment were interpolated, although this step was not performed for electrodes in the regions of interest, and no more than 4 electrodes were interpolated. Signals were resampled to 512 Hz offline, filtered from 0.1 to 30 Hz, and referenced to the average of all electrodes. Ocular artifacts were corrected on scalp electrodes using Independent Component Analysis. ERP waveforms were time-locked to the onset of the target stimuli and segmented from 100 ms prestimulus to 600 ms poststimulus, and a baseline correction of −99.61 ms was applied. Trials with artefacts of eyeblinks and eye movements were semiautomatically deleted by using a threshold of ±50 µV on the EOG electrodes. With the remaining trials, average HEOG difference waveforms in response to left- and right-side distractor presentations were scored to analyse systematic deviations of eye position, which would indicate a slight tendency for the eyes to move in the direction of the distractor. As residual HEOG deviations did not exceed 4 µV, residual HEOG artifacts unlikely survived EOG exclusion criteria. Trials with other artefacts on the scalp electrodes were deleted automatically using a threshold of ±80 µV, gradient of ±75 µV, and low signal change of 0.01 µV. On average, 89% of trials were kept. More trials were removed for distractor absent condition; however, this is likely because targets would appear in lateral regions in this condition (compared with the vertical midline in the remaining conditions), resulting in more lateral eye-movement artefacts as participants identified the target (refer to supplementary materials for more details).

N2pc/P_D_ was measured time-locked to the no distractor and lateral distractor search display (refer to Fig. 1) at electrodes PO7/PO8 where the signal was maximal during the 180–260 ms time period. The selected electrodes and timeframe align with previous literature on the N2pc/P_D_ components (Corriveau et al., 2012; Burra et al., 2017; Dell’Acqua et al., 2006; Eimer & Kiss, 2007; Lucky & Hillyard, 1994a, b). Electrodes contralateral to the stimuli of interest were subtracted from electrodes ipsilateral to the stimuli of interest (i.e., contralateral – ipsilateral). Thus, when the stimulus of interest was on the right-side of space, electrode PO7 was subtracted from electrode PO8, and vice versa for stimuli on the left-side of the space. Left and right presentations were then averaged together for each condition.

## Results

### Valence and intensity ratings

**Fig. 2:**
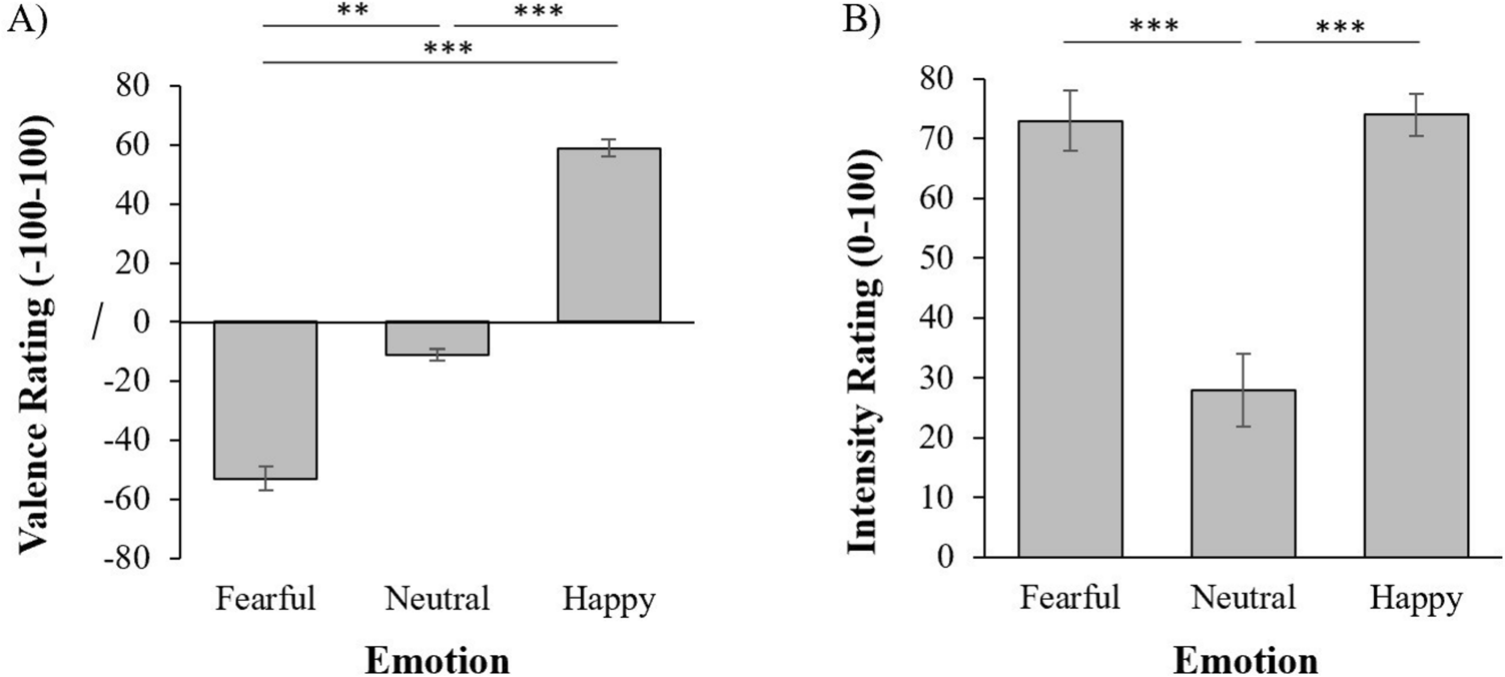
**A** Mean valence ratings. **B** Mean intensity ratings. Error bars represent standard error of the mean. ** *p* <.01, *** *p* <.001

Mean scores of valence and intensity per facial expression were analysed in a one-way, repeated-measures ANOVA (emotion: neutral, fearful, and happy). The mean valence and intensity scores for each emotion are shown in Fig. 2. Mauchly’s test of sphericity was applied to valence ratings and the assumption of sphericity was not met (*p* =.001). Accordingly, we applied the Greenhouse-Geisser correction. A main effect of emotion was found, *F*(1.42, 38.37) = 429.69, *p* <.001, *η*_*p*_^*2*^ =.92. Follow-up frequentist *t*-test revealed that fearful faces (*M* = −51.6, *SD* = 21.3) were rated as more negative than neutral faces (*M* = −11.0, *SD* = 10.9), *t*(27) = −10.95, *p <*.001, and happy faces (*M* = 58.0, *SD* = 13.8), *t*(27) = −18.70, *p* <.001. Additionally, happy faces were rated as more positive than neutral faces, *t*(27) = 17.94, *p* <.001. This was corroborated by Bayesian *t-*test—where the alternative hypothesis was a difference between conditions—with Bayes factor suggesting extreme evidence for the alternative hypothesis across all comparisons (BF_10s_ > 100). With respect to absolute values, there was no difference between fearful and happy faces, *t*(27) = 1.81, *p* =.082, with a Bayes factor suggesting anecdotal evidence for the null (BF_10s_ = 0.8). For the intensity measure, the assumption of sphericity was met (*p* =.179). The one-way repeated measures ANOVA revealed a main effect of emotion, *F*(2, 54) = 110.87, *p* <.001, *η*_*p*_^*2*^ =.80. Follow-up *t-*test revealed that neutral faces (*M* = 28.2, *SD* = 16.3) were perceived as significantly less intense compared with happy (*M =* 74.6, *SD* = 9.2) and fearful faces (*M* = 73.3, *SD* = 13.3); *t*(27) = −12.74, *p* <.001 and *t*(27) = −11.26, *p <*.001, respectively, with Bayes factor for both comparisons suggesting extreme evidence for the alternative hypothesis (BF_10s_ > 100). Importantly, there was no significant difference in intensity between happy and fearful faces, *t*(27) =.445, *p* =.660, with Bayes factor suggesting moderate evidence for the null hypothesis (BF_10s_ = 0.2).

### Behaviour

Only correct responses were included in the analysis. Trials with RTs less than 200 ms and greater than 1,500 ms and RTs exceeding three standard deviations above/below the mean were considered outliers and excluded (an average of 7.4% of the total data). A one-way repeated-measures ANOVA (distractor-presence: absent, fearful, and happy) assessing the differences between the presence of a distractor was conducted. For reaction times, there was a main effect of distractor-presence, *F*(2, 54) = 31.68, *p* <.001. Follow-up pairwise frequentist and Bayesian *t-*tests revealed that RTs were shorter in the distractor-absent trials (778 ms) compared with fearful distractor-present trials (797 ms), *t*(27) = −5.38, *p* <.001, and happy distractor-present trials (801 ms), *t*(27) = −7.33, *p* <.001, with Bayes factor indicating suggesting extreme evidence for the alternative hypothesis (BF_10s_ > 100). However, RTs between happy and fearful distractor-present trials did not significantly differ, *t*(27) = 1.59, *p* =.123, with Bayes factor of 0.6 indicating evidence for the null hypothesis. With respect to accuracy, there was a main effect of distractor-presence, *F*(2, 54) = 3.90, *p* =.026. Follow-up *t*-tests revealed that accuracy was higher in distractor-absent trials (91.1%) compared with fearful distractor-present trials (90.1%), *t*(27) = 2.26, *p* =.032, and happy distractor-present trials (90.1%), *t*(27) = 2.42, *p* =.022, with Bayes factor indicating anecdotal evidence for the alternative hypothesis (BF_10s_ = 1.8–2.3); however, accuracy scores between fearful and happy distractor conditions did not differ, *t*(27) = −0.09, *p* =.929, with Bayes factor of 0.2 indicating evidence for the null hypothesis.

Furthermore, a 2 (emotion: happy, fearful) x 2 (expectation: expected, unexpected) repeated measures ANOVA was performed to assess whether RTs and accuracies changed according to the type of distractor; however, no main effects or interactions reached significance (*Fs <* 2.53, *ps* >.123). We additionally ran a blockwise comparison to increase our sensitivity as effects may be attenuated by repeated exposure of the stimulus. We collapsed our 20 blocks into 5 to ensure that an adequate number of trials comprised an average for each participant for each block. For both RTs and accuracy, we ran a 5 (block: 1, 2, 3, 4, 5) x 2 (emotion: happy, fearful) x 2 (expectation: expected, unexpected) repeated measures ANOVA. Only a main effect of block was present for RTs, and post-hoc *t-*test revealed that RTs were significantly slower in block 1 compared with block 3, 4, and 5 (*ts* > 3.54, *ps* <.004). This is likely indicative of a training effect. No other main effects or interactions were present for accuracy or RTs (*Fs* < 3.10, *ps >*.090).

### Lateralised ERP components

**Fig. 3:**
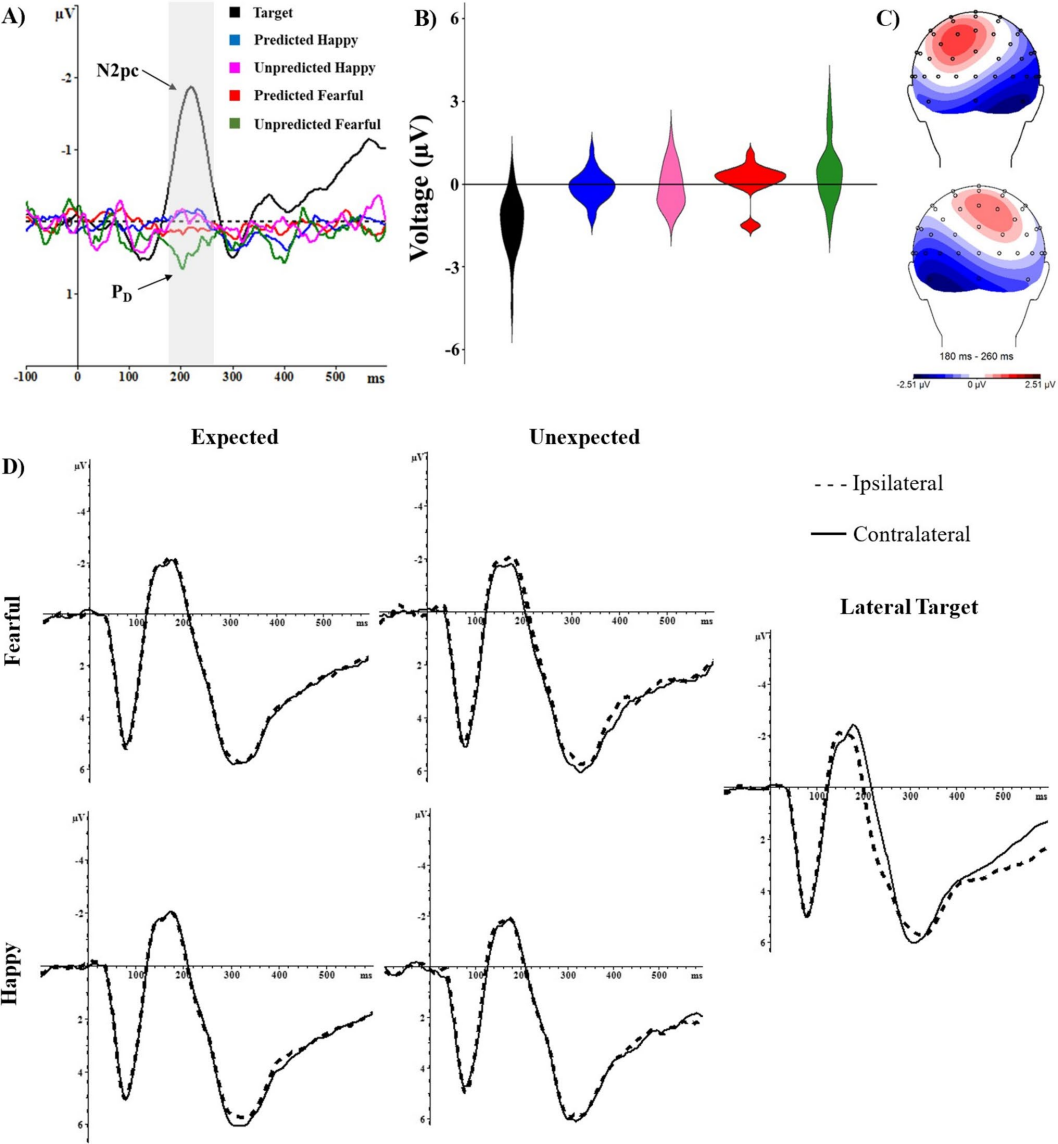
**A** Difference waves of contralateral-ipsilateral activity at PO7/PO8 electrodes for each condition. The gray area indicates the analysis window, around the peak of the N2pc to the target from 180–260ms. **B** Violin plots of target and distractor conditions. **C** The scalp topography of the N2pc. The above topography represents target on left visual field and below topography represents target on right visual field. **D** Ipsilateral and contralateral ERPs at PO7/PO8 electrodes for each condition

A 2 (emotion: happy, fearful) x 2 (expectation: expected, unexpected) repeated measures ANOVA examined whether P_D_ amplitudes differed across distractor types. There was a significant main effect of emotion, *F*(1, 27) = 5.47, *p* =.027, *η*_*p*_^*2*^ = 0.17, with more positive amplitudes for fearful (*M* = 0.3, *SD* = 0.5) than happy faces (*M* = −0.1, *SD* = 0.5). The interaction between emotion and expectation was not significant, *F*(1, 27) = 0.70, *p* =.409, *η*_*p*_^*2*^ = 0.03. However, as Fig. 3 suggested that the main effect of emotion might be driven by the unexpected fearful condition, frequentist and Bayesian one-sample *t*-tests were conducted to assess whether each condition differed from zero, confirming whether a N2pc/P_D_ component was present/absent for each condition. The Bayesian tests used a Cauchy (0, 0.707) prior.

A significant N2pc was present for lateral targets (*M* = −1.4, *SD* = 1.0) when the distractor was absent, *t*(27) = −7.38, *p* <.001, BF_10_ = 2.38 × 10^5^, indicating extreme evidence for the alternative hypothesis. Unexpected fearful distractors (*M* = 0.4, *SD* = 1.0) produced a significant

P_D_, *t*(27) = 2.07, *p* =.048, BF_10s_ = 1.27, providing anecdotal evidence for the alternative hypothesis. However, no other condition differed reliably from zero: expected happy (*M *= −0.1, *SD* = 0.5), *t*(27) = −0.96, *p* =.344, BF_10_ = 0.31; unexpected happy (*M* = −0.1, *SD* = 0.7), *t*(27) = −0.46, *p* =.646, BF_10_ = 0.22; and expected fearful distractors (*M* = 0.1, *SD* = 0.6), *t*(27) = 0.95, *p* =.350, BF_10_ = 0.30, all indicating moderate evidence for the null hypothesis. This suggests that only unexpected fearful distractors elicited a P_D_. To assess whether P_D_ amplitudes changed across the experiment due to habituation, the data were divided into first- and second-half blocks for both expected and unexpected conditions. This analysis revealed no main effect of half and no interaction with expectation (Fs < 1.44, ps >.24). To address the imbalance in trial numbers between expected and unexpected conditions, the data were reanalysed after matching trial counts; the pattern of results remained unchanged (see supplementary materials).

We further conducted targeted paired-sample comparisons to examine whether unexpected and/or expected fearful faces produced larger P_D_ amplitude compared with the happy conditions. This revealed that unexpected fearful faces elicited larger P_D_ amplitudes than both expected, *t*(27) = 2.41, *p* =.012, BF_10_ = 4.50, and unexpected happy faces, *t*(27) = 1.89, *p* =.035, BF_10_ = 1.80, with comparisons providing anecdotal-moderate evidence for the alternative hypothesis. Furthermore, expected fearful faces did not produce significantly larger P_D_ amplitudes than expected, *t*(27) = 1.23, *p* =.115, BF_10_ = 0.56, or unexpected happy faces *t*(27) = 1.05, *p* =.151, BF_10_ = 0.69, with both comparisons showing anecdotal evidence for the null. Overall, these findings suggest that the main effect of emotion was driven by differences between unexpected fearful distractors, despite the absence of a significant interaction in the ANOVA.^1^

Finally, we ran an exploratory analysis correlating P_D_ amplitudes with behavioral measures. For the expected/unexpected happy and expected fearful conditions no correlations

were statistically significant (*ps >*.233). In the unexpected fearful condition, although there was no significant correlation between P_D_ amplitudes and RT (*p* =.913), P_D_ amplitudes were significantly negatively correlated with accuracy scores, *r*(27) = −0.431, *p* =.022. However, this correlation does not sustain significance after a Bonferroni correction for eight comparisons (α =.006).

## Discussion

We investigated how expectations effect the attentional capture/suppression of fearful and happy distractors in a visual search task. Congruent with prior research, participant’s accuracy reduced and reaction times slowed in the presence of distracting facial expressions, but no behavioural differences were observed according to the type of facial expression (Burra et al., 2016, 2017; Hodsoll et al., 2011; Huang et al., 2011). Surprisingly, expectations did not affect participants accuracy/RT to the target and blockwise comparisons highlighted that expectations or facial expressions did not interact with block number for accuracy/RT. However, electrophysiological data revealed that attentional filtering was triggered only for fearful faces when they were unexpected. Specifically, the P_D_ was observed in response to unexpected fearful distractors, but not for expected fearful or expected/unexpected happy distractors. These novel findings emphasise the significant role of expectations in threat detection and subsequent attentional suppression.

## The P_D_ component and relative saliency

The P_D_ component has been found when stimuli situated in the visual field that are not the primary focus of the task but possess features that capture attention nevertheless (see review

by Luck et al., 2021). It is therefore thought to reflect the suppression of salient-but-irrelevant stimuli which compete for attentional selection (Hickey et al., 2009; Mcdonald et al., 2013; Sawaski & Luck, 2010). However, debate has surrounded the time-course of the P_D_ and its relationship with the N2pc. Chen et al. (2023) proposed a three-stage model of attentional response to distractors, whereby attentional capture (signified by an N2pc) occurs before subsequent suppression (i.e., a P_D_). Yet, the saliency of said distractor can qualify this effect, i.e., distractors with lower saliency can elicit a P_D_ without an N2pc (Burra et al., 2017; Chen et al., 2023; Kiss et al., 2012; Zhang et al., 2024). For example, Zhang et al. (2024) demonstrated that the relative saliency between distractor and target effects the attentional processing of distractors. For example, when the relative salience between distractor and target is “low,” the distractor can be directly ignored without the need for attentional processing (i.e., neither an N2pc or P_D_ is elicited); yet when the relative saliency is “medium,” the distractor may be directly suppressed (i.e., eliciting a only P_D_ within or after the time-window of an N2pc); and finally, when relative saliency is “high,” the distractor first captures attention and then is subsequently suppressed (i.e., an N2pc followed by a P_D_). Accordingly, we will utilise this framework to interpret our and previous findings.

## Attentional capture of unexpected facial expressions depends on their relative saliency

Burra et al. (2017) previously found that angry faces, but not happy faces, elicited a P_D_ when embedded in a search array of neutral faces. This implies that angry distractors occupy a “medium” relative saliency, whereas happy faces are of a “low” relative saliency. Our data indicate that feature-based expectations interact selectively with threat-related facial expression. This is surprising, because stimuli that have a low probability given a distribution of expected stimuli are outliers and thus thought to be more informative and to capture attention (Duncan & Humphreys, 1989; Itti & Baldi, 2009). Congruent with this interpretation, feature- and location-regularities for distractor singletons assist with distractor inhibition, as larger RTs are found for low-probability conditions compared with high-probability conditions (Stillwell et al., 2019; Wang & Theeuwes, 2020), or to random versus constant regularities (Gaspelin & Luck, 2018; van Moorselaar et al., 2021), suggesting greater distraction in unexpected conditions.

However, our findings suggest that the attentional capture of unexpected stimuli depends on their relative saliency. For example, the relative salience of happy faces was “low” such that an infrequent or “surprising” appearances did not increase relative salience sufficiently to engender attentional capture ore engage suppression mechanisms. Yet unexpected fearful faces produced a P_D_ without an N2pc, suggesting that distractors were of a medium relative salience, and thus they required direction suppression in order to ignore and complete the task. Because we did not include an equiprobable control (whereby fearful and happy faces appeared with equal frequency), it is difficult to deduce whether low probabilities increased the relative saliency of fearful faces compared with baseline. Irrespective of this, we can conclude that happy distractors were of lower salience relative to threat-related facial expressions and that unexpectedness alone is insufficient to boost the relative salience of any type of distractor in order to elicit attentional capture mechanisms.

## Expecting threat qualifies the attentional capture of threat

Although unexpected fearful faces elicited attentional suppression mechanisms, expected fearful faces did not. This is analogous to how distractors of low relative saliency operate: the distractor can be directly ignored without need for attentional capture or suppression (Zhang et al., 2024). However, we do not conclude that feature-based expectations effect the saliency of the distractor *per se*; rather, feature-based expectations might engage an attentional control system that allows for efficient *suppression* of expected salient features without the need for reflexive attentional mechanisms (for a review of feature-based expectation, see Summerfield & Egner, 2016). We may conclude that threat-related facial expressions are not sufficiently salient to attract attention involuntary, as appropriate top-down control mechanisms, such as feature-based expectations, can restrict the attentional capture of facial expressions. Though, when the threat-related facial expression is unexpected, it is sufficiently salient to require direct suppression.

## A dual-process model of emotion and attention and underlying neural pathways

Our data are consistent with dual‐process models of attention and emotion, which posit that while the fast bottom‐up pathway is designed to rapidly detect potentially important or dangerous stimuli (Pasley et al., 2004; Sander et al., 2005; Vuilleumier et al., 2001, 2004), a parallel top‐down system can modulate or even suppress this capture (Bishop et al., 2007; Pessoa et al., 2002, 2005; Straube et al., 2007; Van Rullen, 2006). For example, visual information is thought to be processed through the cortical retinogeniculo-striate pathway, starting from retina to the lateral geniculate nucleus and then proceeding to visual areas V1–V4 and middle temporal areas; however, in conjunction, a rapid subcortical pathway has been proposed, which may carry specific visual information relating to threat from retina to the amygdala and subsequently to extrastriate visual cortices via retrograde feedback projections from the amygdala (Bertini et al., 2016; Morris et al., 1999; Vuilleumier et al., 2004).

Research demonstrates that lateralized N170 enhancements to fearful faces relies on the integrity of the right amygdala (Framorando et al., 2021). Previously, we found that unattended unexpected fearful faces enhance N170 amplitudes (Chalk & Pegna, 2024). Thus, it is plausible that when an unexpected fearful face appears in a spatially unattended region, this visual information is rapidly processed by subcortical networks and excitatory projections from the amygdala act as an alarm system (resulting in an enhanced N170; Chalk & Pegna, 2024). However, when the threat is irrelevant to the individual’s goals, active attentional suppression occurs to avoid an unwarranted shift in attention (as indexed by a P_D_ 200 ms onwards). Yet, in expected contexts, higher-order cortical regions—particularly those within prefrontal cortex—may activate suppression templates based on statistical learning, effectively downgrading the impact of incoming threat signals, potentially via feedback loops form prefrontal cortex to extrastriate visual areas (Cisler & Koster, 2010; Cosman et al., 2018; Ferrante et al., 2023; Noudoost & Moore, 2011). This proactive inhibition means that the stimulus does not evoke the pronounced alarm signal typically observed for unexpected threats (Chalk & Pegna, 2024). Instead, its processing is dampened, and the need for reactive suppression is reduced or even bypassed.

## Interaction between expectations and threat changes according to task-relevancy in visual search designs

On the surface, our findings appear to challenge research which demonstrates that expectations affect attention towards nonthreatening stimuli but not attention towards threatening stimuli (Aue et al., 2016, 2019; Abado et al., 2023). However, it is important to consider that the task-relevancy of stimuli varies between these studies and our own. In prior studies, participants must actively distinguish threatening from non-threatening stimuli (e.g., spiders from birds) to succeed at the task. Thus, expectations might be unnecessary to avoid situations where an unexpected threat goes unnoticed (Aue et al., 2016, 2019; Abado et al., 2023). However, in our research, the appearance of threat-related facial expressions only intends to hamper an individual at their task. From an evolutionary perspective, top-down expectations might play a crucial role in these situations, as they allow the individual to avoid distraction from a threat that is known and likely been assessed; but a threat which *is not* expected, and is currently irrelevant to the individual’s goals, still requires attentional capture as it signals an alarm to reassess the situation.

## Limitations

First, color singletons significantly dominate the saliency in visual search arrays (Barras & Kerzel, 2017; Zhang et al., 2024). Consequently, this would reduce the relative saliency of facial expression distractors. Thus, it may not be that expected fearful and expected/unexpected happy faces are not salient *per se*, more so that unexpected fearful faces are the most salient type of facial expression relative to the aforementioned facial expressions when facial expressions are paired with a salient colour target. This may explain why we did not find a significant interaction between expectation and facial expression, along with anecdotal evidence for the appearance of a P_D_ for unexpected fearful distractors. Simply, the relative saliency between target and distractors was lower such that fearful faces did not incur a “pop-out” effect which would lead to an N2pc (attentional capture) and stronger P_D_ (attentional suppression; Chen et al. 2023). Future research is necessary to explore the role of relative saliency and its influence on the interaction between expectation and facial expressions on attentional processes.

Second, repetition suppression, which pertains to the reduction of neural activity following repeated stimulus presentations, could partially explain differences in neural activity between high- and low-probability stimuli (Feuerriegel et al., 2021). Although immediate repetition effects may explain our results, we do not believe that large-scale repetition suppression has occurred, because overall, fearful and happy faces were presented at an equal frequency. Additionally, the pattern of our ERP results did not change according to our split-half and trials-matched analyses, and behaviourally there was no interaction between block and expectation. However, we cannot clearly elucidate whether our effects of expectancies are due to mechanisms of predictive processing or surprise: our findings may simply demonstrate a surprise response which is present for unexpected stimuli, not necessarily a suppression affect to expected stimuli that are “silenced” and an enhancement to prediction errors as described in the theory of predictive processing (Friston, 2005; Hohwy, 2020). Furthermore, it may be that the observed effects of expectancy are attributed to an emotional contrast effect or simply that more adaptation takes place for happy compared with fearful faces when they are expected. In either case, fearful faces are more likely to “pop-out” not due to expectation mechanisms but due to contrast or adaption mechanisms. However, again we did not find an interaction between block and emotion for RT or accuracy which may suggest that emotional valence did not change as an account of repeated exposure. Irrespective of these concerns, it is difficult to deny that unexpected fearful faces engender a unique encoding which unexpected happy faces are not privy to (Chalk & Pegna, 2024).

Finally, we find a significant negative correlation between P_D_ amplitudes and accuracy scores, which may suggest that the degree of attentional suppression needed on the distractor fearful face may influence accuracy to the target. However, we are cautious to overinterpret this result as the analysis is exploratory and significance does not withstand when Bonferroni corrections are applied. However, future research should explore how the correlation between P_D_ amplitudes and accuracy to target is affected by the saliency of distractors.

## Conclusion

Overall, our results suggest that feature-based expectations modulate the attentional capture of threat-related distractors. However, unexpectedness alone does not elicit attentional capture of distractors as the effects did not extend to happy distractors. Consequently, it appears that the human attentional system prioritizes threat-related faces; yet this system is malleable to the expectations of the individual: when one can expect threat, it no longer captures our attention. These results provide caveats to *the threat-capture hypothesis*, suggesting that although threat uniquely captures our attention, this may be suppressed with appropriate top-down controls.

## Supplementary Information

Below is the link to the electronic supplementary material.

Supplementary file1 (DOCX 328 KB)

## Data Availability

All data is available at OSF | Active Suppression only occurs for Unpredicted Fearful Faces.

## Code availability

All Psychopy and JASP code is available at OSF | Active Suppression only occurs for Unpredicted Fearful Faces.
