# Supplementary material for: Turning a blind eye: The struggle to inhibit attention towards unexpected negative emotions
Source: Cogn Affect Behav Neurosci. 2026 Mar 18;26(4):1728–40. doi: 10.3758/s13415-026-01415-3 (PMC13385269; doi:10.3758/s13415-026-01415-3)
Supplement: Supplementary file 1 — Supplementary file1 (DOCX 328 KB) [file 13415_2026_1415_MOESM1_ESM.docx]

**First Half (FH) vs. Second Half (SH)**

First half vs. second half analyses were only preformed on the expected emotion. Analyses were not performed on unexpected emotion due to low trial count (first half vs. second half would result in ~ 30 trials for each condition for unexpected trials).

**First Half Repeated Measures ANOVA**

**
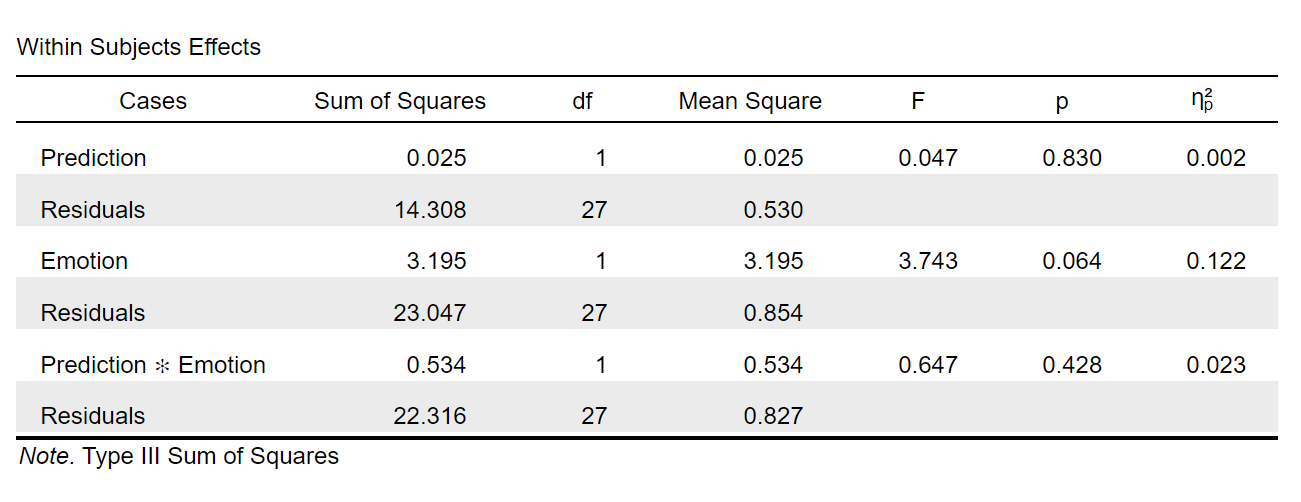
**

**First Half One Sample T-Test Different From Zero**

**
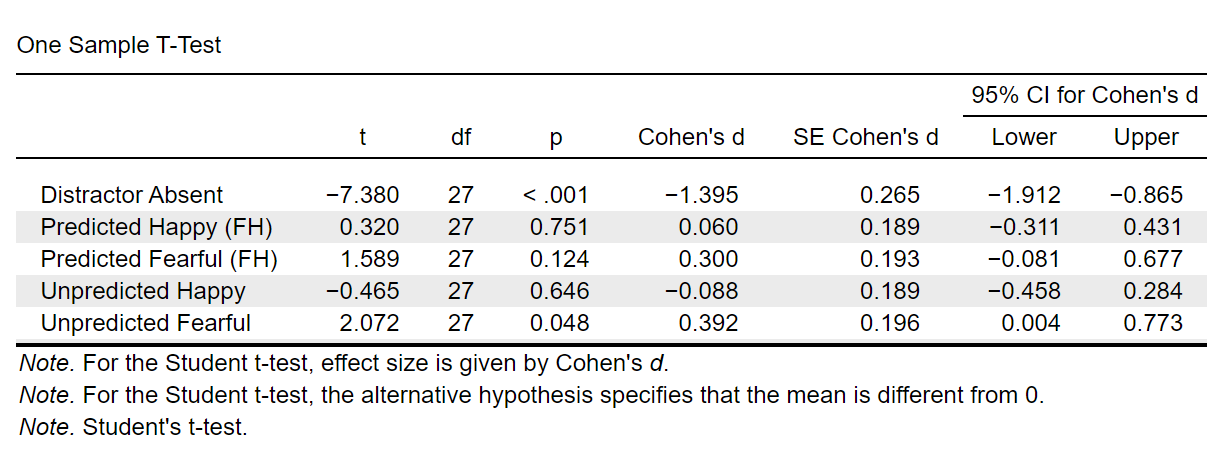
**

**Second Half Repeated Measures ANOVA**

**
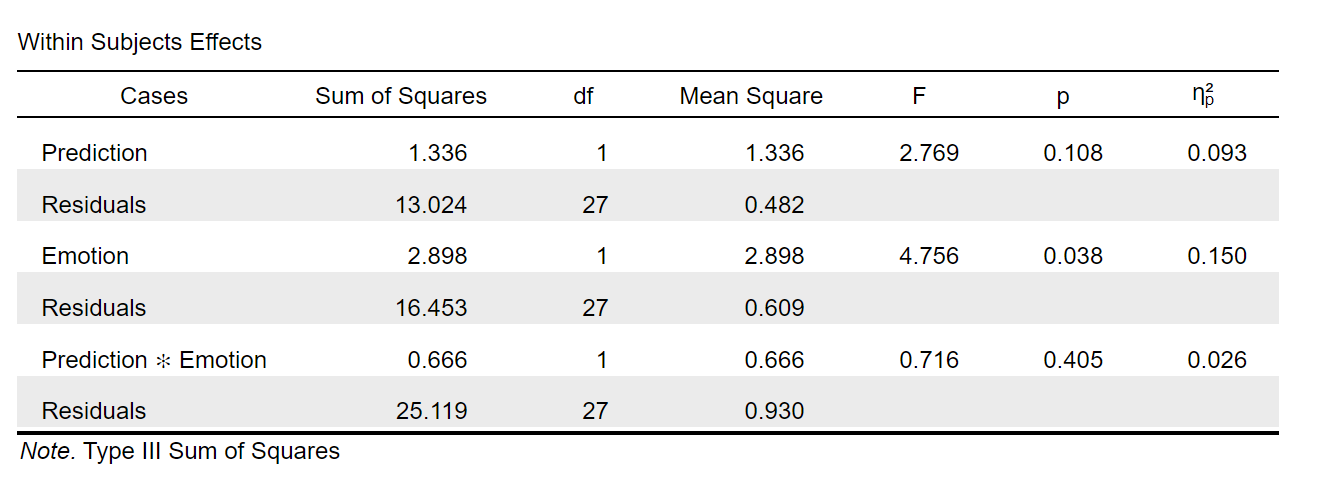
**

**Second Half One Sample T-Test Different From Zero**

**
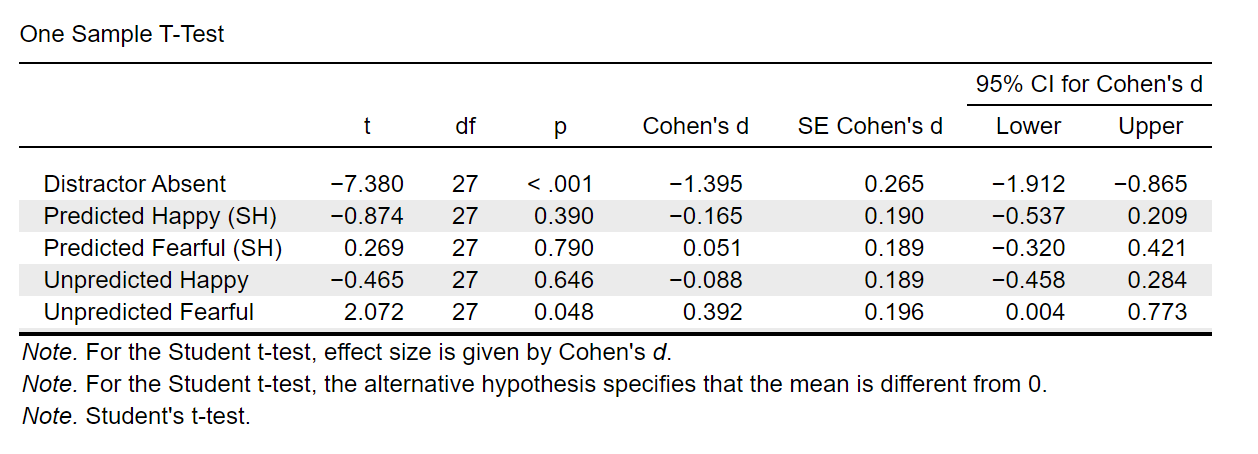
**

**Trials Matched Analyses**

In this analysis, only 70 trials are chosen from the expected condition. These are chosen at random.

**Trials Matched Repeated Measures ANOVA**

**
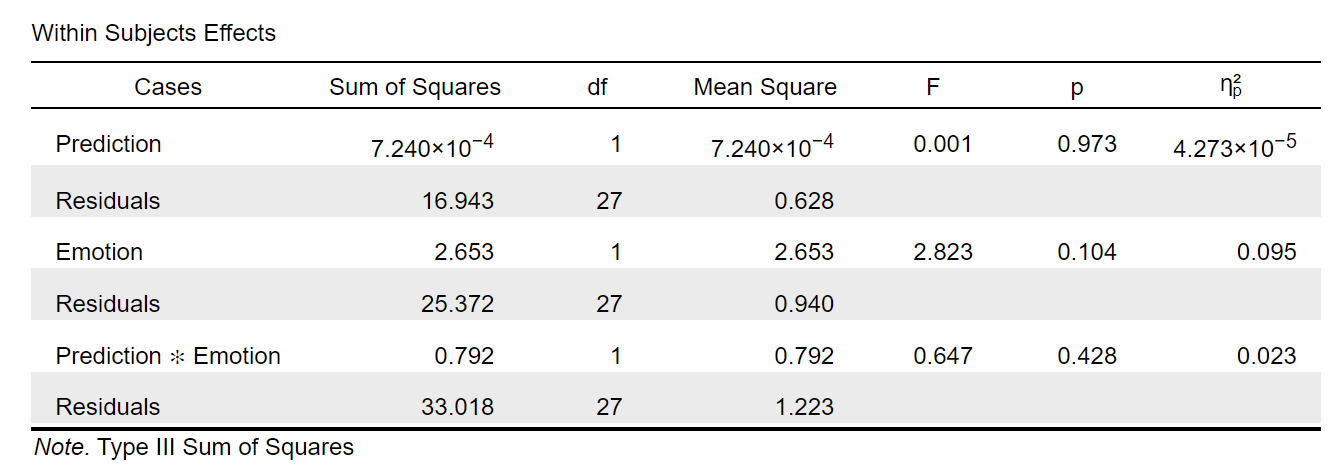
**

**Trials Matched One Sample T-Test Different From Zero**

**
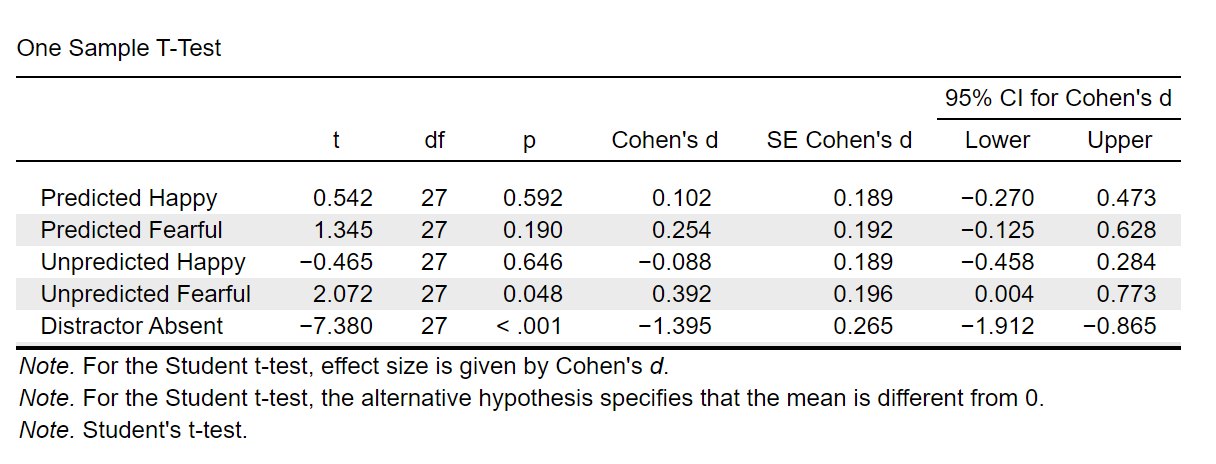
**

**Average Trials Removed**

**Table 1**

*The average proportion of trials removed and average number of trials remaining.*

| Condition | Trials Removed (%) | Trials Remaining |
| --- | --- | --- |
| Distractor Absent  Predicted Happy  Predicted Fearful  Unpredicted Happy | 0.15 (0.11)  0.09 (0.06)  0.10 (0.06)  0.10 (0.07) | 541.9 (69.6)  218.8 (14.5)  216.9 (14.8)  71.9 (5.4) |
| Unpredicted Fearful | 0.11 (0.07) | 71.3 (5.8) |

*Note*. Standard deviation appears in the parentheses next to the means.

**Analysis with Participant 14 (50-year-old) removed**

| **One Sample T-Test** | | | | | | | | | | | | | | | |
| --- | --- | --- | --- | --- | --- | --- | --- | --- | --- | --- | --- | --- | --- | --- | --- |
|  | | | | | | | | | | | | **95% CI for Cohen's d** | | | |
|  | | **t** | | **df** | | **p** | | **Cohen's d** | | **SE Cohen's d** | | **Lower** | | **Upper** | |
| Predicted Happy |  | -0.939 |  | 26 |  | 0.822 |  | -0.181 |  | 0.194 |  | -0.498 |  | ∞ |  |
| Predicted Fearful |  | 1.009 |  | 26 |  | 0.161 |  | 0.194 |  | 0.194 |  | -0.127 |  | ∞ |  |
| Unpredicted Happy |  | -0.458 |  | 26 |  | 0.675 |  | -0.088 |  | 0.193 |  | -0.405 |  | ∞ |  |
| Unpredicted Fearful |  | 1.924 |  | 26 |  | 0.033 |  | 0.370 |  | 0.199 |  | 0.039 |  | ∞ |  |
| Distractor Absent |  | -7.241 |  | 26 |  | 1.000 |  | -1.394 |  | 0.270 |  | -1.832 |  | ∞ |  |
|  | | | | | | | | | | | | | | | |
| *Note.*  For the Student t-test, effect size is given by Cohen's *d* . | | | | | | | | | | | | | | | |
| *Note.*  For the Student t-test, the alternative hypothesis specifies that the mean is greater than 0. | | | | | | | | | | | | | | | |
| *Note.*  Student's t-test. | | | | | | | | | | | | | | | |

| **Within Subjects Effects** | | | | | | | | | | | | | | | | |
| --- | --- | --- | --- | --- | --- | --- | --- | --- | --- | --- | --- | --- | --- | --- | --- | --- |
| **Cases** | | | **Sum of Squares** | | **df** | | | **Mean Square** | | **F** | | | **p** | | **η²_p_** | |
| Prediction | |  | 0.606 |  | 1 | |  | 0.606 |  | 1.389 |  | | 0.249 |  | 0.051 |  |
| Residuals | |  | 11.344 |  | 26 | |  | 0.436 |  |  |  | |  |  |  |  |
| Emotion | |  | 3.157 |  | 1 | |  | 3.157 |  | 5.036 |  | | 0.034 |  | 0.162 |  |
| Residuals | |  | 16.298 |  | 26 | |  | 0.627 |  |  |  | |  |  |  |  |
| Prediction ✻ Emotion | |  | 0.383 |  | 1 | |  | 0.383 |  | 0.530 |  | | 0.473 |  | 0.020 |  |
| Residuals | |  | 18.809 |  | 26 | |  | 0.723 |  |  |  | |  |  |  |  |
|  | | | | | | | | | | | | | | | | |
| *Note.*  Type III Sum of Squares | | | | | | | | | | | | | | | | |
| *Marginal Means - Emotion* | | | | | | | | | | | | | | | | |
|  | | | | **95% CI for Mean Difference** | | | | | |  | |  |  |  |  |  |
| **Emotion** | | **Marginal Mean** | | **Lower** | | | | **Upper** | | **SE** | |  |  |  |  |  |
| Happy |  | -0.082 |  | -0.298 | |  | | 0.134 |  | 0.108 |  |  |  |  |  |  |
| Fearful |  | 0.260 |  | 0.044 | |  | | 0.476 |  | 0.108 |  |  |  |  |  |  |
|  | | | | | | | | | | | |  |  |  |  |  |

| **Bayesian One Sample T-Test** | | | | | |
| --- | --- | --- | --- | --- | --- |
|  | | **BF₁₀** | | **error %** | |
| Predicted Happy |  | 0.304 |  | 0.029 |  |
| Predicted Fearful |  | 0.323 |  | 0.029 |  |
| Unpredicted Happy |  | 0.224 |  | 0.029 |  |
| Unpredicted Fearful |  | 1.008 |  | 0.024 |  |
| Distractor Absent |  | 139246.921 |  | 4.594×10^-10^ |  |
|  | | | | | |
| *Note.*  For all tests, the alternative hypothesis specifies that the population mean differs from 0. | | | | | |

| **Paired Samples T-Test** | | | | | | | | | | | |
| --- | --- | --- | --- | --- | --- | --- | --- | --- | --- | --- | --- |
| **Measure 1** | |  | | **Measure 2** | | **t** | | **df** | | **p** | |
| Unpredicted Fearful |  | - |  | Unpredicted Happy |  | 1.762 |  | 26 |  | 0.045 |  |
| Unpredicted Fearful |  | - |  | Predicted Happy |  | 2.255 |  | 26 |  | 0.016 |  |
| Predicted Fearful |  | - |  | Unpredicted Happy |  | 1.088 |  | 26 |  | 0.143 |  |
| Predicted Fearful |  | - |  | Predicted Happy |  | 1.254 |  | 26 |  | 0.111 |  |
| Unpredicted Fearful |  | - |  | Predicted Fearful |  | 1.054 |  | 26 |  | 0.151 |  |
|  | | | | | | | | | | | |
| *Note.*  For all tests, the alternative hypothesis specifies that Measure 1 is greater than Measure 2. For example, Unpredicted Fearful is greater than Unpredicted Happy. | | | | | | | | | | | |
| *Note.*  Student's t-test. | | | | | | | | | | | |

| **Bayesian Paired Samples T-Test** | | | | | | | | | |
| --- | --- | --- | --- | --- | --- | --- | --- | --- | --- |
| **Measure 1** | |  | | **Measure 2** | | **BF₊₀** | | **error %** | |
| Unpredicted Fearful |  | - |  | Predicted Happy |  | 3.421 |  | ~ 9.856×10^-5^ |  |
| Unpredicted Fearful |  | - |  | Unpredicted Happy |  | 1.500 |  | ~ 1.903×10^-5^ |  |
| Predicted Fearful |  | - |  | Predicted Happy |  | 0.726 |  | ~ 4.189×10^-5^ |  |
| Predicted Fearful |  | - |  | Unpredicted Happy |  | 0.589 |  | ~ 2.699×10^-5^ |  |
| Unpredicted Fearful |  | - |  | Predicted Fearful |  | 0.565 |  | ~ 2.325×10^-5^ |  |
|  | | | | | | | | | |
| *Note.*  For all tests, the alternative hypothesis specifies that Measure 1 is greater than Measure 2. For example, Unpredicted Fearful is greater than Predicted Happy. | | | | | | | | | |
